# Social Media and the Olympics: A Chance for Improving Gender Equality

**DOI:** 10.3389/fspor.2022.825440

**Published:** 2022-04-27

**Authors:** Aneta Grabmüllerová

**Affiliations:** Department of Leadership and Organization, School of Communication, Leadership and Marketing, Kristiania University College, Oslo, Norway

**Keywords:** social media, Olympics, gender equality, National Olympic Committees, Tokyo 2020 Olympic Games

## Abstract

The purpose of this paper is to explore whether social media content by the National Olympic Committees (NOC) during the 2020 Tokyo Olympic Games strengthens or weakens the International Olympic Committee's (IOC) gender equality ambitions. As the media play an important role in creating the impressions that people cherish during and after the Olympics, the IOC has since the 1990s increased its responsibility for fair media portrayal of athletes and competitions by revising its own media production. In the past decade, this most notably concerns social media. Not only has it become an inseparable part of global sports consumption, but it is also seen as a tool for changing the biased and stereotypical portrayal of female athletes in news media, even though male and female athletes have become nearly equal in numbers of participants. Studies of media production and equality-informed decisions are, however, rare in sport. Drawing upon a quantitative analysis of social media accounts of three National Olympic Committees (NOC) (Norway, Czech Republic and Switzerland) and qualitative in-depth interviews with key informants—NOCs' and European Olympic Committee's (EOC) social media personnel—this study therefore explored the decisions and processes that influenced gender portrayal during the 2020 Tokyo Olympics. Findings of the study showed that media personnel have a significant influence on gender portrayal in their respective communication channels. In contrast to news media, they were aware of the frames they apply, and they applied them in alignment with the Olympic values. Consequently, they set a fairer agenda for both male and female athletes and strengthened the gender equality mission of the IOC.

## Introduction

Ever since the invention of sport, women have been in a minority in terms of participation, and their underrepresentation, marginalization and repression has been mirrored in media coverage. Some of the reasons for this are that traditional media coverage is based on values that reproduce traditional conceptions of gender and national identity (Crossan et al., 2021) and it has been oblivious to female athletes (Fink, 2015). Only women's success in mega-sport events such as the Olympic Games has disrupted this practice (Arth et al., 2018). Every other year, billions of eyes are on the biggest sporting event in the world, observing how national pride and values at times override gender (Billings and Angelini, 2007).

With the advent of digital media in the previous decade, sports coverage evolved into an interpersonal, multicultural, and worldwide public realm (Creedon, 2014). Some scholars expected that this development would result in more equitable coverage for female athletes (LaVoi et al., 2019). However, initial studies suggest that old patterns of media representation are being reproduced (LaVoi and Calhoun, 2014; Adá-Lameiras and Rodríguez-Castro, 2021). To rectify this flaw, the IOC has been transformed into a large producer, rather than a conveyor, of media content. This allows them to create their own image rather than rely on news media. It operates the Olympic Channel, an over-the-top Internet television service, and several social media accounts with millions of followers. For example, on Instagram the IOC is followed by an audience of 5.2 million users. Similarly, each of the National Olympic Committees (NOC) runs its own account. How are these media outlets used for promoting the IOC's vision for increased gender equality? Recently, the IOC updated their Portrayal Guidelines for gender balanced representation (IOC, 2014) and its partnership with UN women resulted in a major review project on gender equality. The result of this project are 25 action-oriented recommendations, with three focusing on balanced portrayal of both genders.

Due to the organizational commitment of the 206 NOCs and the European Olympic Committee (EOC) to support IOC's policies and be loyal to the latter's visions, they represent an important intermediary with national audiences. But how do NOCs translate this commitment in terms of producing gender-balanced content on social media? Sports coverage has been described as a hierarchical institution dominated by men with minimum coverage dedicated to female athletes (Cooky et al., 2015). To some extent, this has been caused by the patriarchal environment and hegemonic values in sports newsrooms (Hardin and Shain, 2005). Previous research revealed that journalists are influenced by their personal attitudes to female sport (Organista et al., 2021) and gender distribution of produced news is based on those attitudes (Billings, 2009). Furthermore, they justify women's underrepresentation by their masculine audience assumptions (Dashper, 2018).

Media research has also massively focused on newspapers coverage despite the prevalence of digital media formats (Geurin and Naraine, 2020). Against this backdrop this article contributes to the field by addressing an underexplored issue. Whereas there are many studies of media coverage related to the Olympics (Grabmüllerová and Næss, 2022) only a handful of studies focus on the production of Olympic media content. The research questions in this article are, consequently, threefold:

- What is the gender distribution in social media posts on NOCs' Instagram's accounts?
- Does the number of posts devoted to men and women on social media of Norwegian, Czech and Swiss NOCs correspond with participation and performance?
- How is the commitment of the Olympic movement to gender equality and women's empowerment reflected in NOCs' social media communication?

To answer these questions, this article couples theoretical perspectives on agenda setting and framing decisions with a mixed-methods approach based on social media content analysis and semi-structured interviews. The analysis demonstrated how the commitment of the Olympic movement to gender equality and women's empowerment are reflected in their social media communication. The main findings show that personal influence had a significant influence on the provided content. However, unlike the news media (Organista et al., 2021), the media personnel identified themselves with the Olympic values and commitment to gender equality and women's empowerment. Consequently, it resulted in fairer portrayal of female athletes. This demonstrated how important a role organizational culture plays, a finding that can serve as a guide to news media houses that aim to improve gender equality in sports media coverage.

### Theoretical Framework

In recent years, a vast and expanding framing-theory literature has arisen from a variety of disciplines (Borah, 2011). Its application in media and communication studies has emerged from sociological perspective and is now perceived as a construct of social reality. On the one hand, media frames reality in a way that is comprehensible and predictable (McQuail, 2010). On the other hand, the audience has its own individual frames that help them to process information (Entman, 1993). Media producers may be influenced by social-structural, organizational or individual values and beliefs that leads them to select some aspects of perceived reality in order to make them more salient in communication (Entman, 1993). At the media level, frames then facilitate how media producers organize big amounts of information and deliver them to audiences. They can employ certain linguistic choices and highlight some aspects of reality while excluding others and/or impose a causal theme on their stories (Scheufele, 1999). Framing is a crucial aspect in understanding how the media establish relevance and importance (Fink and Kensicki, 2002).

Together with agenda setting, framing theory explains what role media plays in creation of public meaning. While framing theory describes how information is communicated, agenda setting theory is concerned with what topics are given space in media and to what extent. Agenda setting is based on the strong connection between the importance that mass media put on certain issues and the significance given to these issues by mass audiences (McCombs and Shaw, 1972). In this way, media can systematically affect how the audience perceives events (Price et al., 1997). Both theories have been widely used by scholars examining Olympic media coverage (Billings and Eastman, 2003; Billings et al., 2010; Eagleman, 2015) and sport media (Wensing and Bruce, 2003; Eagleman, 2011). In the past decades, scholars have mostly examined media frames and the consequences of framing but very little research has been dedicated to how are these frames produced (Carragee and Roefs, 2004; Borah, 2011). As Scheufele (Scheufele, 1999) noted, framing is a complex process involving frame (agenda) building, frame setting, individual-level effects of framing; and a link between individual frames and media frames. Geurin and Naraine (Geurin and Naraine, 2020) point out the lack of research concerned with the 'human elements of Olympic media' as there is a lack of examination of social actors, including politicians, organizations and social movements who determine and influence media creation. This is therefore a gap that needs to be filled, especially if we consider that ‘*media has strong, long-term effects on audiences, based on the ubiquitous and consonant stream of messages they present to audiences*' (Scheufele and Tewksbury, 2007, p. 10). Ambivalent sports media coverage of female athletes, as defined by Duncan and Hasbrook (Duncan and Hasbrook, 1988), has a negative consequence on public meaning and society and continues to obstruct the advancement of women's sport. As Bruce (Bruce, 2013) further develops, media coverage of sport matters because it tells us who and what is important, and in what way.

To connect the theoretical framework with the case presented in this study, gatekeeping theory helps explore how and why certain pieces of information pass through so-called gates. Gatekeeping is a process of ‘*selecting, producing, transmitting, and shaping information*' that occurs on multiple levels in various networks of people who produce sports media (Creedon, 2014, p. 17). It's the gatekeepers who decide who and what is valued, relevant and interesting enough to be given attention (LaVoi et al., 2019). The common lack of knowledge of women's sports might cause more generic and plain coverage (Dashper, 2018; Xu, 2019) and therefore reinforce the male domination of sports media production and consumption. Hence, it is crucial to further investigate the role of those actors in framing processes.

### Literature Review

Scholars continually report on sportswomen's underrepresentation in news media (Mesnner et al., 1993; Eastman and Billings, 1999; Eagleman, 2015; Litchfield and Kavanagh, 2019). Their findings indicate that female athletes receive less airtime (Eastman and Billings, 1999; Billings, 2007, 2008) and less space in newspapers (Duncan, 1990; Lee, 1992; Pratt et al., 2008). Gender portrayal is also related to the gender of the producer (Schoch, 2013), to the cultural repertoires (Benson and Saguy, 2005) and the organizational environment (Silcock, 2002; Pfister, 2010). Furthermore, some have suggested that sports coverage of female and male athletes is not (only) affected by gender but other aspects such their percentage participation (Delorme, 2014), success (Bruce et al., 2010), nationalism, culture (Capranica et al., 2005), and the producers themselves (Hardin, 2005; Xu, 2019). Media also tend to pay more attention to “feminine” sports and ignore female athletes competing in “masculine” events (Vincent et al., 2002; Higgs et al., 2003). Furthermore, female Olympians are portrayed ambivalently (Bruce, 2013) and receive more stereotypical comments and comments about their appearance (Kinnick, 1998; Jones et al., 1999; Shields et al., 2004). Media houses also remain male dominated as they continue to use more journalists, male sources and announcers (Capranica and Aversa, 2002; Tuggle et al., 2002).

During the 2016 Olympics, social media really came to prominence and organizational communication gained in significance as they became an indispensable communication channel providing organizations with their own voice (Litchfield and Kavanagh, 2019). Some hoped that the open online space would challenge male hegemony in sports coverage (Bruce, 2013; LaVoi et al., 2019). However, Jones (Jones, 2013) and LaVoi and Calhoun (LaVoi and Calhoun, 2014) discovered that major public broadcasters continued to underrepresent sportswomen even in online spaces. On the other hand, North American Olympic broadcasters generally framed gender along equitable lines on their social media accounts (Johnson et al., 2020). Together with traditional media, social media provide an important source of information and reference points for its audience (Darnell and Sparks, 2005). This implies that understanding the importance of media-intensive sports events like the Olympics requires a knowledge of the processes by which media texts are formed, as well as the reasons why they are constructed in specific ways.

While the IOC puts great importance on gender equality, it failed to translate its own media recommendations and guidance into practice. (Xu, 2019) analyzed gender portrayal in the Olympic Channel over 16 months from 2016 to 2018 revealing that 60.1 per cent of pictures of news stories were devoted to men with greater focus on masculine sports, and only 39.9 per cent to women. Litchfield and Kavanagh (Litchfield and Kavanagh, 2019) analyzed Twitter accounts of the Australian Olympic team @AUSOlympicTeam and Team GB @TeamGB. Despite some prevailing stereotypes, the British content offered fair representations of genders, but Australian women remained underrepresented.

Compared with the proliferation of media content studies, investigations of media content production processes within the Olympic setting are quite rare. Only three publications were discovered in this literature review. The first two focus on media production processes in news media. O'Neill and Mulready (O'Neill and Mulready, 2015) interviewed UK journalists during the 2012 Olympics. The respondents indicated that sports organizations play an important role in media production and they don't do enough to promote women's sport. As demonstrated on the Simon Whitfield case (Darnell and Sparks, 2005), media exposure play a vital role in the construction of athletes' public image, which can endorse their marketability. At the same time, it is in the media producers' hands to decide to whom they will provide the exposure (MacNeill, 1996).

Billings (Billings, 2009) explored whether NBC Olympic producers and sportscasters make choices that privilege one gender over another. He revealed that the producers felt they had set a fair agenda for both men and women, however, content analysis proved otherwise. Furthermore, they admitted they unintentionally and unconsciously report ambivalently on men and women. With regard to social media, Xu and Billings (Xu and Billings, 2021) contributed to the understanding of production of digital media in a sports organization committed to gender equality and women's empowerment. By interviewing the media professionals working at the Olympic Channel, they discovered that individuals and their personalities have a significant influence on news production.

Notwithstanding the recent emergence of social media studies in relation to the Olympics, we know very little about how these platforms affects content production processes and existing content analyses of social media yielded mixed results. To address this gap, this study aims to explore how gender is constructed on social media accounts of the Olympic Movement. To that end, the next section will introduce the materials and methods used.

### Methodology

Although many studies have examined gender distribution in coverage of major sports events, few studies have used mixed methods to explain the ongoing underrepresentation of female athletes. To fill this void, a mixed-methods approach was applied to this study. The reason was twofold. First, it provides credible and usable knowledge by combining and integrating quantitative and qualitative methodologies (Molina-Azorin and Fetters, 2019). By combination of different perspectives on social phenomena, mixed methods contribute to broader and deeper understanding of them (Hollstein, 2014). Such an approach enables a comparison with and contrast to the amount of content given and provides reasoning behind it. Secondly, while it has been commonly employed by social media researchers (Snelson, 2016), it has not yet been used to study production of the Olympic social media content.

A mixed-methods approach is here understood as “*the combination of methodologies in the study of the same phenomenon*” (Denzin, 1978, p. 291). Ideally, this means that “*the bias inherent in any particular data source, investigators, and particularly method will be canceled out when used in conjunction with other data sources, investigators, and methods*” (p. 14); and that “*the result will be a convergence upon the truth about some social phenomenon*” (p. 14). Denzin (1978), moreover, outlines the emergence of three possible outcomes from triangulation: convergence, inconsistency, and contradiction. Which of these outcomes characterized this study, and how the integration of different data types was analyzed, typically a conflict theme in methodology debates (Johnson et al., 2007), will be explained below. But first, the rationale for each method and corresponding data must be introduced (Table 1).

**Table 1:** Analyzed accounts.

**Instagram accounts**	**Number of followers**
	**(October 2021)**
Czech Olympic committee @olympijskytym	179 k
Swiss Olympic committee @swissolympicteam	51.2 k
Norwegian Olympic committee @olympicteamnorway	20.2 k

#### Method 1: Content Analysis

Content analysis of social media network Instagram was employed. This analysis served to answer the first two research questions regarding the gender distribution and its connection to participation and performance. Instagram was used because it had the highest engagement rate^1^ across social media networks used by the selected NOCs. To select relevant data, a non-probability judgmental (purposive) sampling method was used. Following Blaikie & Priest (2019), this method is optimal for selecting some cases of particular type in order to study some aspects of organizational behaviors. In such a case, the selection of the sample is a matter of judgements, which may be informed by theoretical considerations. For the purpose of this study, three European countries with diverse national identity, culture, economy, language, and sport habits were selected. Those countries are also situated in different parts of the scale of the World Economic Forum's Global Gender Gap report (World Economic Forum, 2021). The selection includes Norway, a country with long-term high ranking, the Czech Republic, a post-communist country that continuously ranks low, and Switzerland, a country that was one of the last in Europe to recognize women's rights, yet nowadays sits in the upper part of the scale. Such studies can focus and compare different aspects of individual attitudes, values and beliefs, and aspects of organizations, institutions and structures (Blaikie and Priest, 2019).

The data collection was conducted during the 2020 Tokyo Olympics that was held from 23rd July to 8th August 2021. Data was recorded manually and analyzed using Microsoft Excel. The data were recorded by the author and then validated by a disinterested but competent peer in the “peer debrief” process (Hail et al., 2011). Peer debriefing is defined as a “*process of exposing oneself to a disinterested peer in a way similar to an analytic session for the goal of examining parts of the inquiry that could otherwise stay simply implicit inside the inquirer's thinking*” (Lincoln and Guba, 1985, p. 308). Statistical software R was used to analyze the data, calculate descriptive statistics and tests (Spearman correlation coefficients, tests of significance of the correlation coefficient and tests of independence). Categorization was adopted from the Portrayal Guidelines for gender-equal, fair, and inclusive representation in sport published by the IOC (IOC, 2014). “Visibility” category evaluating who is featured in the content was limited to: number of posts, type of post (picture or video), sport, and gender. Data in each category was compared to gender, participation, and medal success and used to establish a standard of comparison for quantity of media content.

#### Method 2: Semi-structured Interviews

Semi-structured interviews were of use to answer the third research question the commitment of the Olympic movement to gender equality. Combining then the semi-structured interview method with quantitative measures allowed the former to bring nuance and depth to the latter's panoramic qualities. What is characteristic about the interviewee sample in this article's topic is that it is only a handful of persons that are responsible for social media in NOCs and the EOC. Given that “*the content of inquiry is such that complete or in-depth information cannot be expected from representative survey respondents*” (Kumar et al., 1993, p. 1634), the four informants included here are thus considered “key informants”. These informants “*serve as gatekeepers regulating access to people and information and as cultural experts explaining culture to an outsider*” (McKenna and Main, 2013, p. 116). As this research had an exploratory dimension (Blaikie and Priest, 2019), allowing participants to give freely their own experiences and feelings regarding their work habits, performances and management styles, the composition of the sample is crucial (Crouch, 2006).

Four interviews were conducted with media personnel of three European NOCs and the EOC with the aim to extract professional experiences and insights and gain understanding of their working processes. Individuals representing the organizations were asked to report on their activities as they occurred in their natural settings. Following Blaikie and Priest (Blaikie and Priest, 2019), four main kinds of data of individuals' characteristics were collected: demographic characteristics, knowledge, attitudes, and reported behavior. Respondents were contacted and recruited by the researcher through email and LinkedIn. All respondents were anonymized. Three interviews took place from June to September 2021 around the time of the Tokyo Olympics, and one interview was conducted earlier as part of different but relevant study. Due to the pandemic, all the interviews were conducted via Zoom or Teams (based on respondent's preference). All the respondents were white, in their young adulthood, educated and had several years of experience in the field. Two respondents were women, two were men.

Literature on content production in sports and discussion with media experts preceded the development of the interview guide, which started with general and non-controversial questions (e.g., what is your job title?). After rapport was established, more probing questions were asked (e.g., to what extent do you think the NOC fulfills its promises in promoting gender equality?). After transcribing the interviews verbatim, thematic analysis was employed to interpret the data and understand how social media personnel constructed athletes' identity during the Tokyo Olympics. Themes and patterns of meaning within the data that were related to the research question were identified by employing inductive and theoretical thematic analysis (Braun and Clarke, 2013). Thematic textual analysis is a systematic and repeatable approach for analyzing texts. It is a method of deciphering the meanings of text to comprehend their greater cultural relevance. To evaluate the collected data, qualitative data analysis tool NVivo was used. Following this (Braun and Clarke, 2006), transcripts were reviewed to gain a sense of the information gathered. Then, the transcripts were processed to extract patterns and code the data. These codes were then re-examined and grouped into themes. Each theme was identified and named and is represented by quotes in the results section.

### Results: Content Analysis

The content analysis revealed the gender distribution on the social media accounts on the Czech, Norwegian and Swiss Olympic Committee's Instagram accounts. A total of 432 posts were analyzed. To provide a context, the participation and medal success of the teams are presented in Table 2.

**Table 2:** Number of participants, medals, and posts.

	**Czech republic**		**Norway**		**Switzerland**	
	**Female**	**Male**	**Female**	**Male**	**Female**	**Male**
Number of participants	41 (35.7%)	74 (64.3%)	34 (36.6%)	59 (36.6%)	47 (44%)	60 (56%)
Number of medals	2 (18.2%)	9 (81.1%)	1 (12.5%)	7 (87.5%)	10 (76,9%)	3 (23.1%)
Number of posts	56 (36.1%)	99 (63.9%)	29 (34.9%)	54 (65.1%)	45 (65.1%)	30 (40%)

The Tokyo Olympics was ‘first ever gender-balanced' with overall participation at 48.8 per cent women and 51.2 per cent men. All the three analyzed teams were represented by a similar number of athletes (CZE: 115, SUI: 107, NOR: 93). The Czech team consisted of 64.3 per cent men and 35.7 per cent women, similarly the Norway ratio was at 63.4 per cent and 36.6 per cent, respectively. Swiss representation was 44 per cent women and 56 per cent men. In terms of success, the Czechs won 11 medals, 9 of them by men, 2 by women. Similarly, Norwegian men won 7 out of 8 medals for Norway. Switzerland received 13 medals, 10 of them won by women. Participation and performance figures provide a standard of comparison for quantity of media content.

The number of posts was analyzed based on participation proportion and success. In the case of the Czech Republic and Norway, the gender distribution of posts corresponded with the participation ratio. In the case of Switzerland, women received a higher number of posts (60 per cent). However, that can be explained by their medal success as they won most medals for Switzerland (76.9 per cent). In all three cases, media content was related to athletes' participation by sport. The Spearman correlation indicated that there is an association between sport participation and the amount of social media content (CZE: r_s_ =0.534, *p* = 0.01; SUI: r_s_ = 0.904, *p* ≤ 0.001; NOR: r_s_ = 0.615, *p* = 0.01). In the case of the Czech Republic and Norway, this association increased where the athlete is female; the more women participate in each sport at the Olympics, the more content they will receive (CZE: male: r_s_ = 0.445, *p* = 0.04; female: r_s_ = 0.591, *p* = 0.004; NOR: male: r_s_ = 0.571, *p* = 0.03; female: r_s_ = 0.801, *p* < 0.001). On the other hand, in the case of Switzerland, the association increased when the athlete was male (male: r_s_ =0.936, *p* < 0.001; female: r_s_ =0.733, *p* < 0.001).

In all three cases, the correlation of medal success and media content was significant for both male and female athletes (CZE: r_s_ = 0.928, *p* < 0.001; NOR: r_s_ = 0.937, *p* < 0.001; SUI: r_s_ = 0.814, *p* < 0.001). Sports in which athletes won medals resulted in more content. However, it's not only success that influences the amount of content but also its expectation and structural advantage of some sports. Sports that resulted in medal success received most of the posts in both Norway and the Czech Republic. This was especially true for Czech female athletes as only nine posts (16 per cent) were dedicated to sports, in which female athletes did not succeed. On the other hand, for the Swiss Olympic Committee medal success was not decisive. More posts were dedicated to sports that did not result in medal success.

### Results: Semi-structured Interviews

Even though the quantitative and qualitative data collection was ongoing simultaneously, the qualitative interviews served an explanatory and validatory role (Johnson et al., 2007) in this study. As Johnson, Onwuegbuzie, and Turner (Johnson et al., 2007) state, “during the data analysis stage, qualitative data can play an important role by interpreting, clarifying, describing, and validating quantitative results, as well as through grounding and modifying.” Through this process, not only the third research question was addressed but also convergence of the two data sets was identified. In all three studied cases, content analysis revealed fair amount of content dedicated to female athletes with focus on both: their participation and success. This was stressed by the respondents:

“The whole coverage is based on our shared beliefs of being one team. We don't distinguish between sports or athletes; instead, we aim to cover all types of sports.”

At the same time, one respondent described the additional content dedicated to successful athletes:

“We had graphics with a medal or position during the Olympic Games, but we didn't prepare it just for the medalists; we had it for top 10 as that is still a wonderful accomplishment for us.”

Reflecting on the quantitative findings, three main principles were identified through the qualitative interviews: individual agency (the role of gatekeepers), living up to Olympic values (Organizational role), and social responsibility.

#### The Role of Gatekeepers

Given that one of the IOC's key goals is to promote gender equality, it is not unexpected that the Olympic values have been embedded in the NOCs' communication. Media personnel of the NOCs expressed consensus that both men and women should receive equitable amount of content. The Olympic representative very specific about how Olympic values play an important role in their work:

“The coverage of the Olympic movement and its sports is our primary aim. Our fundamental concept is that we are one team, one sport, and our aim is to encourage a healthy lifestyle and sports participation in everyday life.”

And how they promote them despite the possible lower engagement:

“We have projects that are important to us as an organization and we see that there is a good reaction to it that encourages to do more, but we also have Olympic values content that is maybe not so popular, but we still like to do it because it's important to us. Like this we can implement it in the population or at least in the sport.”

This seemed to have a direct influence on the equality in the portrayal:

“We are really conscious when it comes to diversity. We want to portray both female and male athletes, to cover the broad range of different athletes and different sports that are out there.”

However, in some cases the respondents found it difficult as some sports and attire are more revealing:

**“**We avoid sexual connotation, pictures or anything like that but it's difficult in some sports like beach volleyball. Some sports naturally put the body in focus, but I think we are pretty sensitive to the topic.”

Notwithstanding the fact that they claimed to have considerable autonomy to express their individual personalities and opinions in their reporting, they stressed the importance of collaboration with colleagues:

“It's mostly my decision but if it's a controversial image, I ask someone else if this is okay, but I think we really try to show the sports.”

“Because the material is created by a large group of individuals, my job is to ensure that everything satisfies those specifications [of our identity]. I'm the one who approves it and decides whether or not it should be published.”

Furthermore, they explained the importance of balanced mixed gender teams in the work processes:

“It also helps that I'm a woman myself. I don't have a different view on it [women's and men's performance] and that's what's great with our department that consists of both men and women. We have those different perspectives, and we can always challenge each other when it comes to portrayal of athletes. I think it's our strength. We live up to the fact that we see them as athletes and it's not dependent on gender.”

All the three NOCs had mixed gender teams, and some stressed how their personalities and experiences had a positive impact on gender equality in the social media content:

“Maybe it's also personal because I was working with para-athletes before and there the pictures are even more important and sometimes it's sensitive pictures. So, I think I am a bit more aware what an image can do.”

#### Organizational Role

The IOC recently published Portrayal guidelines “*recognizing that sports coverage is very influential in shaping gender norms and stereotypes*” with the aim to “*raise awareness and call for gender-equal and fair representation of sportspeople across all forms of media and communication*” (IOC, 2014). This seemed to have a great influence on the quality of the content, even if some of the NOCs did not follow specifically the IOC's guidelines but had their own internal rules.

“I think it's really important [the guidelines] and from our side, we follow these guidelines. I try to find a good balance between male and female portrayal also in our newsletter, on our website, or social media posts. We try, we are careful about this topic, we work hand-in-hand with the IOC. So basically, we follow the principles.”

Despite the document's recommendatory character, it seemed to also exercise a controlling effect:

“I think the IOC does have supervisory body during the games and I don't know which kind of system they use to catch the wrong message or the wrong attitude but since they have the guidelines that should be respected, I suppose that they do have some system to oversee this activity.”

On the other hand, they also mentioned how diverse structure of the Olympic events play role in the post's distribution:

“We want to maintain our communication continuous during the Olympics since certain events, endure for several days while some other sports have only a one-day competition. All of this must be considered.”

It is part of the IOC's Gender review project (IOC, 2018, p. 8) to have an “*equal representation of women's and men's events in the competition schedule*” and to ensure the competition formats to be equal in the distances, duration of competition segments, number of round etc. However, this goal hasn't been met yet.

#### Social Responsibility

Sports journalists previously justified men's domination of media coverage by financial profit and denied the responsibility of promoting women's sports (Vincent et al., 2003; Knoppers and Elling, 2004). In comparison to news media, the IOC's own media production is not a primary activity or source of finance. Media personnel of the NOCs do not therefore have to follow that logic:

“I think that there is a difference in between the traditional media and the Olympic movement. I see a lot the different points of view. We don't focus just on the consumption but also on the values.”

“We are not run by engagement and likes and that popularity contest that is already out there. For us, it's never been a big goal to grow or get a ton of engagement on each post.”

Most coverage that athletes receive is controlled by mass media, however, social media let athletes create their own portrayal. For athletes, social media provide a way to connect with fans, stakeholders, and sponsors. They can be especially beneficial for athletes from sports who usually do not receive mainstream coverage as social media is a tool to build and promote their own personal brand (Eagleman, 2013). While mass media ignore athletes' preferences of portrayal, media personnel of the NOCs took that into consideration:

“If we have a campaign and I use an athlete's picture, I ask if they are okay with that picture. Like now with the Olympic Games, we want to ask everyone if the photo is okay.”

“We always want to please the athlete, even though we don't need their consent to work with their pictures.”

“I'm not in direct contact with the athletes but a lot of us are, and if we use pictures that are not okay, then we will have a bad reaction from them directly.”

Geurin-Eagleman and Burch (Geurin-Eagleman and Burch, 2016) explained that it is NOCs' interests to develop strong relationships with their athletes as it helps to create a positive impression of both the athletes and the sports organizations. Furthermore, athletes' self-representation also influences the reputation of their respective sports organizations. One of the respondents explained how this is executed:

“[My daily routine] includes keeping the relations with athletes…how should they communicate or what they should post, or if they want and advice–they come to us.”

Geurin-Eagleman and Burch (Geurin-Eagleman and Burch, 2016) further explained that while NOCs cannot control athletes' social media accounts, they should develop a strong understanding of effective social media practices to further an athlete's desired long-term brand image. Ultimately, this a mutual relationship—NOCs influence the audience's perception of the athletes and the other way round.

## Discussion

The first two research questions were: (1) What is the gender distribution in NOCs' social media communication? and (2) Does the number of posts devoted to men and women on social media of the Norwegian, Czech and Swiss NOCs correspond with participation and performance? The content analysis showed equitable social media content in all three countries reflecting athletes' participation and performance. While in the case of the Czech Republic and Norway, success was a more prominent factor, Switzerland dedicated content to all participants regardless of performance. In comparison to previous studies that continuously drew attention to sportswomen's underrepresentation in sports coverage, the three NOCs provided fair amount of content dedicated to both female and male athletes. These results demonstrated that if media content is analyzed with consideration of participation and national success, masculine hegemony, and cultural preferences of certain sports disappears.

Even though medal success increased the number of posts dedicated to particular sports, less successful participants also received a proportional amount of content, and this applied to both genders. Seemingly equally important was the expectation of success, which bears out some of the previous studies (Bruce et al., 2010; Ličen and Billings, 2013; Crossan et al., 2021). Athletes that were favorites in their disciplines received more national coverage with no regard to their gender. At the same time, some sports are more complicated than others and have a structural advantage that results in more coverage (Wehden et al., 2019). Some events differ in length and advance structure as men's events tend to be longer and/or include more teams. Together with national success in these events, it naturally leads to more coverage.

From the perspective of framing and agenda setting, it is understood that media play a vital role in creation of public meaning. Therefore, fair portrayal of female athletes has an important impact on the consumer's perception of sport and its medialization. However, this study is limited to frame (agenda) building and frame setting. While the literature documenting gender distribution in Olympic coverage has been extensive (Grabmüllerov and Næss, 2022), the actual consequences of gender framing on audience remain understudied. Therefore, the individual-level effects of framing should be explored in future studies. More studies such Metcalfe's (2019) or Jones and Greer's (2012) exploring audience attitudes influenced by media consumption are needed.

In previous studies, sports journalist were described as misogynist and reluctant to cover female athletes claiming they are not responsible for the improvement of gender equality (Knoppers and Elling, 2004; Organista et al., 2021). In other studies, journalists who claimed fair agenda were contradicted by the actual content analysis (Billings, 2009; Xu, 2019). This study showed convergence of the quantitative and qualitative data (Denzin, 1978). Perhaps this is a result of more balanced Olympic content production teams (gatekeepers) comparing to the overly masculine environment of sports journalism (Knoppers and Elling, 2004). From the framing and agenda setting perspective, social media personnel consciously decided to cover athletes based on their participation in the Olympics and their success. Agenda and frames were described as result of collective work, however, at the end of the media process was one gatekeeper who had a significant individual influence on the final outcome and consequently gender portrayal and distribution in their respective communication channel. As described earlier, those gatekeepers decide who and what receives attention (LaVoi et al., 2019). NOCs' media personnel were conscious about setting a fair agenda for both male and female athletes and aware of the frames they apply.

This answers the third research question: How is the commitment of the Olympic movement to gender equality and women's empowerment reflected in NOCs' social media communication? Loyalty to the Olympic movement and its values seemed to be vital to set a fair agenda in the content production. Social-structural or organizational characteristics, as well as individual or ideological variables, may influence producers' framing of an issue at the media level (Scheufele, 1999). During the interviews with the NOCs' media personnel, it became clear that this is the case. Organizational and personal dedication to gender equality was the key aspect. In this regard, it can be argued that the increased women's visibility in the social media is influenced by the IOC's gender equality policies and organizational context of the Olympic movement.

On the other hand, the cultural context did not influence the production or opinions on gender equality. Despite the different nations in this study, the same attitudes were identified in the interviews and female athletes were fairly represented in all the analyzed accounts. This can be explained by the commitment of the NOCs and their personnel to the IOC and Olympic values. While the European cultural context did not have an influence on the amount of content dedicated to female and male athletes, it affects female's involvement in sport in general. Professional sportswomen's participation and success is heavily associated with national gender regimes and women's higher empowerment in a society is reflected in elite sport participation (Meier et al., 2021). Therefore, more localized initiatives to promote gender equality in sport and society are needed. For future studies, more culturally diverse sample would be beneficial as it can provide an understanding of what role ethnicity and race play.

Despite the positive findings within the Olympic movement, outside of the period of major sport events, sportswomen's coverage in news media drops to minimum as one of the respondents mentioned:

“Very impressive figure is that only 4 per cent of the media coverage is dedicated to women in sport. And this is a very sad figure, and we should ask ourselves many questions about why it happens? I think the IOC is committed to this cause.”

The IOC's initiative to increase women's participation and consequently opportunity to succeed is essential as this is mirrored in media coverage. Despite the claimed balanced participation in the Tokyo Olympics (Tokyo IOC, 2020), there are still sports and events that differ for female and male athletes. For example, men's football started with 16 teams while the women's tournament had only 12 teams. Similarly, ice hockey will feature 12 men's teams but only 10 women's teams in the upcoming Winter Olympics. Differences also remain in individual sports, for example men's longest cross-country skiing event is 50 km while women compete on a 30 km long track. This results in shorter events which can sway the gender distribution of the coverage.

## Conclusion

The objective of this study was to explore the context of Olympic content production and gender distribution. The analysis showed that all three National Olympic Committees offered fair amount of content of female athletes corresponding with their participation and performances. Interviews with the media personnel showed convergence with the content analysis. The set fair coverage was a result of the personal and organizational commitment of the Olympic movement to gender equality and women's empowerment. This is an important finding for the theoretical perspectives of framing, agenda setting and gatekeeping as well as for the IOC and other media producers who wish to improve coverage of female athletes, as it provides an explanation of the contradictory results in many other studies of the Olympic coverage and gender distribution. It also serves as evidence supporting the anticipation that disruptions of the persisting narratives will come from media forms other than sports news or live sport (Bruce, 2013). Social media and other alternative digital spaces might have the power to shift the existing cultural and media discourses (Bruce, 2013; Peeters et al., 2019).

The scope of this study is limited because of its nature as a case study. To expand, the sample consists of diverse demographics, however, it is limited to Europe and countries with fair attitudes toward gender equality. Therefore, only “natural generalizabity” can be considered (Blaikie and Priest, 2019). Furthermore, this research analyzed gender distribution in social media posts only quantitatively. Future research should examine social media content through qualitative media analysis as well as to explore how is such content and its framing interpreted by the consumers. Especially, since Smith Clavio and Lang (Smith et al., 2021) identified the effects of visual framing of athletes on social media. Such studies would help us to better understand the framing effects of ambivalent gender portrayal and the importance of gender equality in sports media.

Despite these limitations, this study contributes to the understanding how social media frames are constructed in the Olympic movement and revealed the influence of gatekeepers in the agenda setting. The implications for further research are that more studies on social media coverage and gender distribution are needed. We can deduce from this study that to improve gender equality in news media, institutional change would be required as well as local initiatives to promote gender equality in society. Media gatekeepers play an important part in it as they have the power to influence the audience's view of social reality by imposing their own perceptions on their media content (Xu and Billings, 2021).

## Data Availability Statement

The raw data supporting the conclusions of this article will be made available by the authors, without undue reservation.

## Author Contributions

The author confirms being the sole contributor of this work and has approved it for publication.

## Conflict of Interest

The author declares that the research was conducted in the absence of any commercial or financial relationships that could be construed as a potential conflict of interest.

## Publisher's Note

All claims expressed in this article are solely those of the authors and do not necessarily represent those of their affiliated organizations, or those of the publisher, the editors and the reviewers. Any product that may be evaluated in this article, or claim that may be made by its manufacturer, is not guaranteed or endorsed by the publisher.

## Footnotes

^1^Engagement rate is calculated as a percentage of interactions per overall number of followers.

## References

[B1] Adá-Lameiras A. Rodríguez-Castro Y. (2021). Analysis from a gender perspective of the Olympic Games on Twitter. Eur. Sport Manag. Q. 0, 1–17. 10.1080/16184742.2021.1910965

[B2] Arth Z. W. Hou J. Rush S. W. Angelini J. R. (2018). (Broad)casting a wider net: clocking men and women in the primetime and non-primetime coverage of the 2018 Winter *Olympics. Commun. Sport*. 7. 10.1177/2167479518794505

[B3] Benson R. Saguy A. C. (2005). Constructing Social Problems in an Age of Globalization: A French-American Comparison. Am Soc. Rev. 10.1177/000312240507000203

[B4] Billings A. C. (2007). From diving boards to pole vaults: gendered athlete portrayals in the “big four” sports at the 2004 Athens Summer Olympics. South. Commun. J. 72, 329–344. 10.1080/10417940701667563

[B5] Billings A. C. (2008). Clocking gender differences: televised olympic clock time in the 1996-−2006 Summer and Winter Olympics. Telev. New Media. 9, 429–441. 10.1177/1527476408315502

[B6] Billings A. C. (2009). Conveying the olympic message: NBC producer and sportscaster interviews REGARDING THE ROLE OF IDENTITY. J. Sports Media. 4, 1–23. 10.1353/jsm.0.0027

[B7] Billings A. C. Angelini J. R. (2007). Packaging the games for viewer consumption: gender, ethnicity, and nationality in NBC's coverage of the 2004 Summer Olympics. Commun. Q.. 55, 95–111. 10.1080/01463370600998731

[B8] Billings A. C. Angelini J. R. Holt Duke A. (2010). Gendered profiles of olympic history: sportscaster dialogue in the 2008 Beijing Olympics. J. Broadcast. Electron. Media.54, 9–23. 10.1080/08838150903550352

[B9] Billings A. C. Eastman S. T. (2003). Framing identities: gender, ethnic, and national parity in network announcing of the 2002 Winter Olympics. J. Commun. 53, 569–586. 10.1111/j.1460-2466.2003.tb02911.x

[B10] Blaikie N. Priest J. (2019). Designing Social Research. 3rd edition. Cambridge, UK: Malden, MA: Polity. p. 352.

[B11] Borah P. (2011). Conceptual issues in framing theory: a systematic examination of a decade's literature. J. Commun. 61, 246–263. 10.1111/j.1460-2466.2011.01539.x

[B12] Braun V. Clarke V. (2006). Using thematic analysis in psychology. Q Res Psychol. 3, 77–101. 10.1191/1478088706qp063oa

[B13] Braun V. Clarke V. (2013). Successful Qualitative Research: A Practical Guide for Beginners. SAGE. p. 402.

[B14] Bruce T. (2013). Reflections on communication and sport: on women and femininities. Commun. Sports. 1. 10.1177/2167479512472883

[B15] Bruce T. Hovden J. Markula P. (2010). Sportswomen at the Olympics. A Global Content Analysis of Newspaper Coverage. Rotterdam: Sense Publisher. 10.1163/9789460911071

[B16] Capranica L. Aversa F. (2002). Italian television sport coverage during the 2000 sydney olympic games: a gender perspective. Int. Rev. Sport Sociol. 37, 337–349. 10.1177/101269020203700309

[B17] Capranica L. Minganti C. Billat V. Hanghoj S. Piacentini M. F. Cumps E. (2005). Newspaper Coverage of Women's Sports During the 2000. Sydney Olympic Games: Belgium, Denmark, France, and Italy. Res Q Exerc Sport. 76, 212–223. 10.1080/02701367.2005.10599282 16128488

[B18] Carragee K. M. Roefs W. (2004). The neglect of power in recent framing research. J. Commun. 54, 214–233. 10.1111/j.1460-2466.2004.tb02625.x33140229

[B19] Cooky C. Messner M. A. Musto M. (2015). “It's Dude Time!”: a quarter century of excluding women's sports in televised news and highlight shows. Commun. Sport. 3, 261–287. 10.1177/2167479515588761

[B20] Creedon P. (2014). Women, social media, and sport*:* global digital communication weaves a web. Telev. New Media. 15. 10.1177/1527476414530476

[B21] Crossan W. Hrebenárová L. Price T. (2021). Windows of equity? Media review of Czech gender hegemony during the 2018 Winter Olympic Games. Acta Gymnica. e2021.004. 10.5507/ag.2021.004

[B22] Crouch M. (2006). McKenzie H. The logic of small samples in interview-based qualitative research. Social Sci. Inf. 45, 483–499. 10.1177/0539018406069584

[B23] Darnell S. C. Sparks R. (2005). Inside the promotional vortex: canadian media construction of Sydney olympic triathlete simon whitfield. Int. Rev. Sport Sociol. 40, 357–376. 10.1177/1012690205059873

[B24] Dashper K. (2018). Smiling assassins, brides-to-be and super mums: the importance of gender and celebrity in media framing of female athletes at the 2016 Olympic Games. Sport Society. 21, 1739–1757. 10.1080/17430437.2017.1409729

[B25] Delorme N. (2014). Were Women Really Underrepresented in Media Coverage of Summer Olympic Games (1984-2008)? An Invitation to Open a Methodological Discussion Regarding Sex Equity in Sports Media. Mass Commun. Society. 17, 121–147. 10.1080/15205436.2013.816740

[B26] Denzin N. K. (1978). The Research Act: A Theoretical Introduction to Sociological Methods. McGraw-Hill. p. 392.

[B27] Duncan M. C. (1990). Sports photographs and sexual difference: images of women and men in the 1984 and 1988 Olympic Games. Sociol. Sport J. 7, 22–43. 10.1123/ssj.7.1.22

[B28] Duncan M. C. Hasbrook C. A. (1988). Denial of power in televised women's sports. Sociol. Sport J. 5, 1–21. 10.1123/ssj.5.1.1

[B29] Eagleman A. (2011). Stereotypes of race and nationality: a qualitative analysis of sport magazine coverage of MLB players. J. Sport Manag. 25, 156. 10.1123/jsm.25.2.156

[B30] Eagleman A. N. (2013). Acceptance, motivations, and usage of social media as a marketing communications tool amongst employees of sport national governing bodies. Sport Manage. Rev. 16, 488–497. 10.1016/j.smr.2013.03.004

[B31] Eagleman A. N. (2015). Constructing gender differences: newspaper portrayals of male and female gymnasts at the 2012 Olympic Games. Sport Society. 18, 234–247. 10.1080/17430437.2013.854509

[B32] Eastman S. T. Billings A. C. (1999). Gender parity in the olympics: hyping women athletes, favoring men athletes. J. Sport Soc. Issues. 10.1177/0193723599232003

[B33] Entman R. M. (1993). Framing: toward clarification of a fractured paradigm. J. Commun. 43, 51–58. 10.1111/j.1460-2466.1993.tb01304.x

[B34] Fink J. S. (2015). Female athletes, women's sport, and the sport media commercial complex: Have we really “come a long way, baby”? Sport Manage. Rev. 18, 331–342. 10.1016/j.smr.2014.05.001

[B35] Fink J. S. Kensicki L. J. (2002). An imperceptible difference: visual and textual constructions of femininity in sports illustrated and sports illustrated for women. Mass Commun. Soc. 5, 317–339. 10.1207/S15327825MCS0503_5

[B36] Geurin A. N. Naraine M. L. (2020). 20 years of olympic media research: trends and future directions. Front. Sports Act. Living. 2, 129. 10.3389/fspor.2020.572495 33345133 PMC7739588

[B37] Geurin-Eagleman A. N. Burch L. M. (2016). Communicating via photographs: A gendered analysis of Olympic athletes' visual self-presentation on Instagram. Sport Manage. Rev. 19, 133–145. 10.1016/j.smr.2015.03.002

[B38] Grabmüllerová A. Næss H. E. (2022). Gender equality, sport media and the Olympics, 1984–2018: an overview, in The Routledge Handbook of Gender Politics in Sport and Physical Activity, 1st edn, eds Bullingham R. Molnar V. (Routledge).

[B39] Hail C. Hurst B. Camp D. (2011). Peer debriefing: teachers' reflective practices for professional growth. Crit. Stud. Educ. 2, 74–83.

[B40] Hardin M. (2005). Stopped at the Gate: Women's Sports, “Reader Interest,” and Decision Making by Editors. Journal. Mass Commun. Q. 82, 62–77. 10.1177/107769900508200105

[B41] Hardin M. Shain S. (2005). Strength in numbers? The experiences and attitudes of women in sports media careers. Journal. Mass Commun. Q. 82, 804–819. 10.1177/107769900508200404

[B42] Higgs C. T. Weiller K. H. Martin S. B. (2003). Gender Bias In The (1996). Olympic Games: A Comparative Analysis. J. Sport Soc. Issues. 27, 52–64. 10.1177/0193732502239585

[B43] Hollstein B. (2014). Mixed methods social networks research: an introduction, in, Hollstein B Domínguez S. editors. Mixed Methods Social Networks Research: Design and Applications. Cambridge: Cambridge University Press. 10.1017/CBO9781139227193.003

[B44] IOC (2014). New IOC guidelines to ensure gender-equal, fair and inclusive representation in sport in Tokyo - Olympic News. International Olympic Committee 2021. Available online at: https://www.olympics.com/ioc/news/new-ioc-guidelines-to-ensure-gender-equal-fair-and-inclusive-representation-in-sport-in-tokyo

[B45] IOC (2018). IOC Gender Equality Review Project. Lausanne, Switzerland: International Olympic Committee. p. 36. Available online at: https://stillmed.olympic.org/media/Document%20Library/OlympicOrg/News/2018/03/IOC-Gender-Equality-Report-March-2018.pdf

[B46] Johnson R. B. Onwuegbuzie A. J. Turner L. A. (2007). Toward a Definition of Mixed Methods Research. J. Mixed Methods Res. 1, 112–133. 10.1177/1558689806298224

[B47] Johnson R. G. Romney M. Hull K. Pegoraro A. (2020). Shared space: how north american olympic broadcasters framed gender on instagram. Commun. Sport. 216747952093289. 10.1177/2167479520932896

[B48] Jones A. Greer J. (2012). Go “Heavy” or go home: an examination of audience attitudes and their relationship to gender cues in the 2010. olympic snowboarding coverage. Mass Sport is Commun. Sport. 15, 598–621. 10.1080/15205436.2012.674171

[B49] Jones D. (2013). Online coverage of the 2008. Olympic Games on the ABC, BBC, CBC and TVNZ. Pacific Journal. Rev. 19, 244–263. 10.24135/pjr.v19i1.248

[B50] Jones R. Murrell A. J. Jackson J. (1999). Pretty versus powerful in the sports pages: print media coverage of U.S. Women's Olympic Gold Medal Winning Teams. J. Sport Soc. Issues. 23, 183–192. 10.1177/0193723599232005

[B51] Kinnick K. (1998). Gender bias in newspaper profiles of 1996 Olympic athletes: a content analysis of five major dailies. Women's Stud. Commun. 21, 212–237. 10.1080/07491409.1998.10162557

[B52] Knoppers A. Elling A. (2004). ‘We Do Not Engage in Promotional Journalism': discursive strategies used by sport journalists to describe the selection process. Int. Rev. Sport Sociol. 39, 57–73. 10.1177/1012690204040523

[B53] Kumar N. Stern L. W. Anderson J. C. (1993). Conducting interorganizational research using key informants. Acad. Manag. Ann. 36, 1633–1651. 10.5465/256824

[B54] LaVoi N. M. Baeth A. Calhoun A. S. (2019). sociological Perspectives of Women in sp—ort. Routledge Handbook of the Business of Women's Sport. Routledge. p. 36–46. Available online at: https://www.taylorfrancis.com/

[B55] LaVoi N. M. Calhoun A. S. (2014). Digital media and women's sport: an old view on “new” media, in Routledge Handbook of Sport and New Media. London: Routledge. p. 320–30.

[B56] Lee J. (1992). Media portrayals of male and female olympic athletes: analyses of newspaper accounts of the 1984 and the 1988 Summer Games. Int. Rev. Sport Sociol. 27, 197–219. 10.1177/101269029202700301

[B57] Ličen S. Billings A. C. (2013). Cheering for ‘our' champs by watching ‘sexy' female throwers: Representation of nationality and gender in Slovenian 2008. Summer Olympic television coverage. Eur. J. Commun. 28, 379–396. 10.1177/0267323113484438

[B58] Lincoln Y. S. Guba E. G. (1985). Naturalistic Inquiry. SAGE. p. 422. 10.1016/0147-1767(85)90062-8

[B59] Litchfield C. Kavanagh E. (2019). Twitter, team GB and the Australian olympic team: representations of gender in social media spaces. Sport Society. 22, 1148–1164. 10.1080/17430437.2018.1504775

[B60] MacNeill M. (1996). Networks: producing olympic ice hockey for a national television audience. Sociol. Sport J. 13, 103–124. 10.1123/ssj.13.2.103

[B61] McCombs M. Shaw D. (1972). The agenda-setting function of mass media. Public Opinion Q. 36, 176–187. 10.1086/267990

[B62] McKenna S. A. Main D. S. (2013). The role and influence of key informants in community-engaged research: a critical perspective. Action Res. 11, 113–124. 10.1177/1476750312473342

[B63] McQuail D. (2010). McQuail′s Mass Communication Theory. SAGE. p. 633.

[B64] Meier H. E. Konjer M. V. Krieger J. (2021). Women in international elite athletics: gender (in)equality and national participation. Front. Sports Act. Living. 3, 241. 10.3389/fspor.2021.709640 34514387 PMC8429847

[B65] Mesnner M. A. Duncan M. C. Jensen K. (1993). Separating the men from the girls: the gendered language of televised sports. Gender and Society. 7, 121–137. 10.1177/089124393007001007

[B66] Metcalfe S. N. (2019). The development of an adolescent sporting gendered habitus: young people's interpretation of UK sports-media coverage of Rio 2016. Olympic Games. Eur. J. Commun. 16, 361–378. 10.1080/16138171.2019.1693145

[B67] Molina-Azorin J. F. Fetters M. D. (2019). Building a better world through mixed methods research. J. Mixed Methods Res. 13, 275–281. 10.1177/1558689819855864

[B68] O'Neill D. Mulready M. (2015). The invisible woman?: a comparative study of women's sports coverage in the UK national press before and after the 2012 Olympic Games. Journal. Pract. 9, 651–668. 10.1080/17512786.2014.965925

[B69] Organista N. Mazur Z. Lenartowicz M. (2021). “I Can't Stand Women's Sports”: the perception of women's sports by polish sports journalists. Commun. Sport. 9, 372–394. 10.1177/2167479519876886

[B70] Peeters R. Elling A. Sterkenburg J. V. (2019). WEURO 2017 as catalyst? The narratives of two female pioneers in the Dutch women's football media complex. Soccer and Society. 20, 1095–107. 10.1080/14660970.2019.1680506

[B71] Pfister G. (2010). Women in sport – gender relations and future perspectives. Sport Society. 13, 234–248. 10.1080/17430430903522954

[B72] Pratt J. Grappendorf K. Grundvig A. (2008). LeBlanc G. gender differences in print media coverage of the 2004 Summer Olympics in Athens, Greece. Women Sport Phys. Act. J. 17, 34–41. 10.1123/wspaj.17.2.34

[B73] Price V. Tewksbury D. Powers E. (1997). Switching trains of thought: the impact of news frames on readers' cognitive responses. Commun. Res. 24, 481–506. 10.1177/009365097024005002

[B74] Scheufele D. (1999). Framing as a theory of media effects. J. Commun. 49, 103–122. 10.1111/j.1460-2466.1999.tb02784.x

[B75] Scheufele D. A. Tewksbury D. (2007). Framing, agenda setting, and priming: the evolution of three media effects models. J. Commun. 57, 9–20. 10.1111/j.0021-9916.2007.00326.x

[B76] Schoch L. (2013). ‘Feminine' writing: the effect of gender on the work of women sports journalists in the Swiss daily press. Media Cult Soc. 35, 708–723. 10.1177/0163443713491300

[B77] Shields S. Gilbert L. Shen X. Said H. A. L. (2004). At print media coverage across four olympiads. Women Sport Phys. Act. J. 13. 87–99. 10.1123/wspaj.13.2.87

[B78] Silcock B. W. (2002). Global news, national stories: producers as mythmakers at germany's deutsche welle television. Journal. Mass Commun. Q. 79, 339–352. 10.1177/107769900207900206

[B79] Smith L. R. Clavio G. Lang A. (2021). Does visual framing drive eye gaze behavior? The effects of visual framing of athletes in an increasingly visual social media world. Media Psychol. 24, 562–579. 10.1080/15213269.2020.1765810

[B80] Snelson C. L. (2016). Qualitative and mixed methods social media research: a review of the literature. Int. Rev. Sport Sociol. 15, 1609406915624574. 10.1177/1609406915624574

[B81] Tokyo IOC (2020). First ever gender-balanced Olympic Games in history, record number of female competitors at Paralympic Games - Olympic News. International Olympic Committee. 2021. Available online at: https://www.olympics.com/ioc/news/tokyo-2020-first-ever-gender-balanced-olympic-games-in-history-record-number-of-female-competitors-at-paralympic-games

[B82] Tuggle C. A. Huffman S. Rosengard D. S. (2002). A descriptive analysis of NBC's Coverage of the 2000. Summer Olympics. Mass Commun. Soc. 5, 361–375. 10.1207/S15327825MCS0503_7

[B83] Vincent J. Imwold C. Johnson J. T. Massey D. (2003). Newspaper coverage of female athletes competing in selected sports in the 1996. Centennial Olympic Games: the more things change the more they stay the same. Women Sport Phys. Act. J. 12, 1–21. 10.1123/wspaj.12.1.1

[B84] Vincent J. Imwold C. Masemann V. Johnson J. T. A. (2002). comparison of selected “serious” and “popular” British, Canadian, and United States newspaper coverage of female and male athletes competing in the Centennial Olympic Games: did female athletes receive equitable coverage in the “Games of the Women”? Int. Rev. Sport Sociol. 37, 319–335. 10.1177/101269020203700312

[B85] Wehden L.-.O Schröer N. (2019). Golden news? Analysis of summarizing coverage of the Olympic Winter Games 2018 on German TV. MJ. 43, 101–121. 10.24989/medienjournal.v43i1.1795

[B86] Wensing E. H. Bruce T. (2003). Bending the rules: media representations of gender during an international sporting event. Int. Rev. Sport Sociol. 38. 10.1177/1012690203384001

[B87] World Economic Forum (2021). Global Gender Gap Report 2021. Geneva, Switzerland: World Economic Forum. p. 405. Available online at: https://www.weforum.org/reports/ab6795a1-960c-42b2-b3d5-587eccda6023/

[B88] Xu Q. (2019). Challenging The Gender Dichotomy? Examining Olympic Channel Content, Production, and Audiences Through A Gendered Lens. Ann Arbor, United States.

[B89] Xu Q. Billings A. C. (2021). Voices of the gatekeepers: examining the olympic channel production through a gendered lens. Mass Commun. Soc. 24, 629–650. 10.1080/15205436.2020.1864650

